# CRISPR-edit point mutant allele detection (CEPAD)-PCR method for rapid screening of CRISPR edited point mutations

**DOI:** 10.17912/micropub.biology.000368

**Published:** 2021-02-17

**Authors:** Kenneth Trimmer, Swathi Arur

**Affiliations:** 1 University of Texas MD Anderson Cancer Center

## Abstract

CRISPR-Cas9 mediated genome editing is widely used for generating genetic lesions in *C. elegans*. Detection of single-site mutations in F1 progeny after CRISPR-Cas9 injections is currently labor intensive due to lack of a single step PCR-based detection method. Here we present CEPAD-PCR, an allele-specific PCR detection method based on generating silent mutations around the site of the desired genetic lesion during the CRISPR-Cas9 genome editing process. Detection of the desired allele is then performed by taking advantage of the tetra primer PCR method, based on the principle described in the ARMS-PCR. In the CEPAD-PCR, however, unlike ARMS-PCR, presence of additional silent mutations near the desired site-specific mutation in the genome results in PCR priming with high specificity resulting in a low false positive rate. As proof of concept, the method was successfully tested on point mutations in two different genes, *daf-15 *and *raga-1*.

**Figure 1. CEPAD-PCR method overview and screening:**
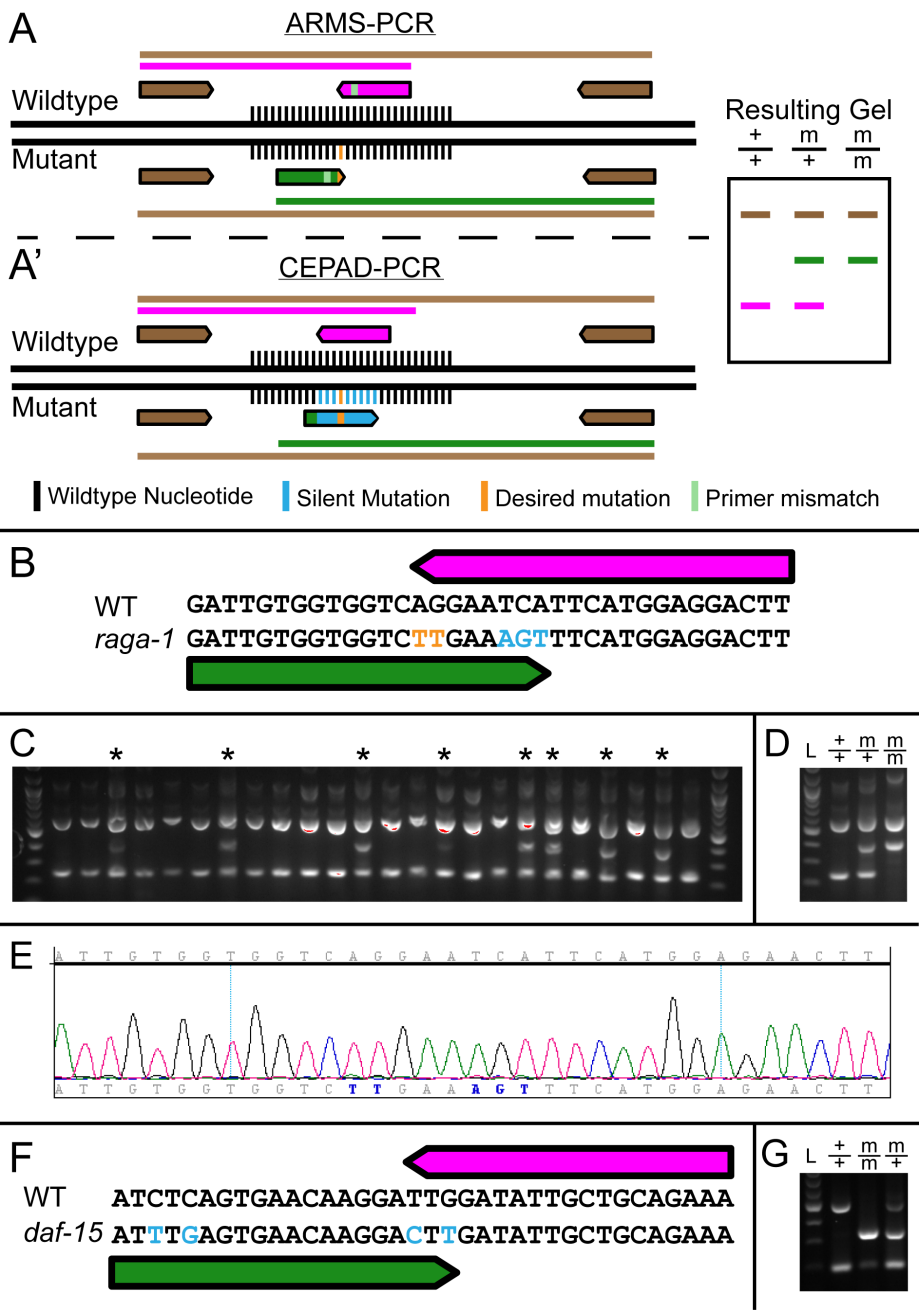
(A, A’) Schematic of ARMS-PCR (A) and CEPAD-PCR (A’) with an expected gel electrophoresis pattern common to both methods. Internal primers which bind to wildtype (pink) or the edited mutant (green) are shown. Brown arrows mark the external primers. Different colors on the gel (right) indicate the specific mutant or wildtype bands with colors being represented by primer pairs. Black nucleotides indicate “unchanged” nucleotides, cyan indicate silent mutations, and orange indicate CRISPR-Cas9 edited mutations. Light green stripe indicates a mismatch only present in the primer. (B) CRISPR-Cas9 edit scheme of *raga-1(Q63L)* mutation with edited (orange) and silent (blue) mutations for CEPAD-PCR. Internal primers for CEPAD-PCR are shown overlapping over the mutated region, the 3’ end of which is on a mutated nucleotide for each. (C) F1 worms screened with CEPAD-PCR. Lanes with an additional band corresponding to the *raga-1(Q63L)* mutant allele are labeled (asterisk). (D) F2 worms screened with CEPAD-PCR. Shown are representative banding patterns for wildtype and *raga-1(Q63L)*, heterozygous and homozygous. (E) Sequencing results for one isolated line. (F) CRISPR-Cas9 edited *daf-15*(*S907AS948A*) allele with silent mutations for removal of a PAM site (blue). Wildtype (reverse, magenta) and mutant (forward, green) primers overlap by 3 nucleotides, two of which are silent mutations. (G) CEPAD-PCR screening for *daf-15*(*S907AS948A*). A faint ghost band corresponding to the wildtype size is present in the homozygous mutant.

## Description

CRISPR has become widespread as a method for generating targeted mutations in *C. elegans*. Screening for introduced point mutations in *C. elegans* is aided by the use of Co-CRISPR strategy, which takes advantage of co‑editing an unrelated locus (such as *dpy-10*) to visibly identify the worms in which at least one efficient Cas9 mediated homologous recombination has occurred (Arribere *et al.* 2014; Kim *et al.* 2014; Paix *et al.* 2015). In most current methods being used, detection of the edit is performed using restriction digestion of a PCR product and sequencing 100-150 potential candidates. To make the process of screening for point mutations more efficient and a one step process (a simple PCR), we have adapted the tetra-primer amplification refractory mutation system (ARMS)-PCR method for use with CRISPR (Sullenberger and Maine 2018). The ARMS-PCR method utilizes 4 PCR primers (Figure 1A), two outer primers at different distances from the point mutation, and two primers in opposing directions which terminate on the point mutation, with a wildtype primer in one direction containing the wildtype nucleotide at its 3’ end, and a mutant primer in the reverse direction containing the mutant nucleotide at its 3’ end (Figure 1A). While this single 3’ mismatch in a primer does not prevent amplification of the incorrect template, an additional mismatch is added to both primers 1-3 nucleotides from the 3’ end to further destabilize the internal primers. This additional mismatch decreases the amplification of the incorrect primer to a greater extent than the correct primer, allowing each primer to only amplify from its respective template. Since the primer binding is unstable, however, the detection rate of false positives and false negatives (due to improper primer destabilization) is relatively high. Significant optimization of PCR conditions, including concentrations of PCR components such as Mg^2+^, is often required to obtain a high level of specificity for amplification of the allele specific product (Medrano and de Oliveira 2014). This degree of PCR condition optimization in the absence of an existing allele is not possible and thus the ARMS-PCR is not useful for screening CRISPR-Cas9 based genome edited mutations. Here we present CEPAD-PCR, an allele-specific PCR detection method based on generating silent mutations around the site of the desired genetic lesion during the CRISPR-Cas9 genome editing process.

In CEPAD-PCR, we introduce silent mutations along with the desired mutation using CRISPR-Cas9. Multiple silent mutations are introduced at the 3’ end of each primer (Figure 1A’). This increases primer binding specificity such that the primers detect the edited allele by sequence specificity alone rather than based on destabilization of primer pairs, requiring very little optimization of PCR conditions. Since contiguous mutations produce higher sequence specificity (Kwok *et al.* 1990; Bru *et al.* 2008), we favor silent mutations which are contiguous with the point mutant. To enable this, we primarily chose leucine, arginine, or serine residues to host the silent mutations since the first nucleotide in each of their codons can be easily changed due to redundancy, allowing for combination with a silent mutation in the previous amino acid to form contiguous mutations. We then deployed the CEPAD-PCR method to screen for single point mutations in *raga-1*.

*raga-1* was CRISPR-edited to changeQ63 to leucine, followed by screening with CEPAD‑PCR using primers which differed by 5 nucleotides total from the wildtype locus (Figure 1B). Detection of the desired edits was performed on candidates using the four primer CEPAD-PCR, with heterozygous edited worms identified by an additional band (Figure 1C). These candidates were isolated, homozygosed, and sequenced to verify that the desired nucleotide edits had occurred (Figure 1D, E). We also tested CEPAD-PCR on a CRIPSR-edited allele which we had previously screened using PCR followed by restriction digest. During backcrossing of the *daf-15*(S907AS948A) CRISPR-edited allele, we designed CEPAD-PCR primers that differ by only 2 nucleotides at the 3’ end yet gave distinct banding patterns for wildtype, heterozygous and homozygous lines (Figure 1F, G).

## Methods

CRISPR

Co-CRISPR design, injection and screening was performed according to Paix *et al.* 2015 (Paix *et al.* 2015) with the addition of silent mutations to allow for CEPAD-PCR-based detection. Likewise, screening was performed by CEPAD-PCR and homozygous F2 isolates were sequenced. All PCR reactions were performed on individual worms using the standard single worm DNA lysis followed by a tetra primer PCR using 2X Hot Start Master Mix Blue, Buffer I (Apex).

CEPAD-PCR primer design and optimization.

Silent mutations were introduced near the desired point mutation to differentiate primer binding to either the wildtype or mutant template as described above. Two internal primers were then designed to overlap the region of differential binding at the 3’ end. Two outer primers were then designed at different distances from the allele-specific primers. All four primers should have the same or similar Tm to ensure that the mutant band will be present under optimized conditions. To optimize the PCR conditions for the CEPAD-PCR, 3 gradient PCR reactions were performed: wildtype primers only, outer primers only, and all primers together. A temperature was chosen at which a single band was visible for wildtype primers and outer primers respectively, together with a wildtype band in the “all primer” condition without extraneous bands at the expected size for the mutant band.

A detailed protocol for the CEPAD-PCR CRISPR edit design, primer design, and primer optimization can be found on our lab website..https://www.mdanderson.org/research/departments-labs-institutes/labs/arur-laboratory/resources.html

## Reagents

**PCR Reagents:**

**Table:** 

Name	Cat#	Source
2X Hot Start Master Mix BLUE, Buffer I	5200600-1250	Apex BioResearch Products

**Strains Used:**

**Table:** 

Strain	Genotype	Designation	Source
AUM1653	*raga-1(viz128)II*	Q63L	This study
AUM1679	*daf-15(viz134viz140)IV*	S907AS948A	This study
